# The latent tuberculosis infection cascade of care in Iqaluit, Nunavut, 2012–2016

**DOI:** 10.1186/s12879-019-4557-3

**Published:** 2019-10-24

**Authors:** Christopher Pease, Alice Zwerling, Ranjeeta Mallick, Mike Patterson, Patricia Demaio, Sandy Finn, Jean Allen, Deborah Van Dyk, Gonzalo G. Alvarez

**Affiliations:** 1 Department of Medicine, The Ottawa Hospital General Campus, 501 Smyth Road, Ottawa, ON K1H 8L6 Canada; 2 Ottawa University School of Epidemiology and Public Health, Ottawa, Canada; 3 Ottawa Hospital Research Institute, Ottawa, Canada; 4 Ministry of Health, Government of Nunavut, Iqaluit, Nunavut Canada; 5 Nunavut Tunngavik Inc, Iqaluit, Nunavut Canada; 6 Ottawa University Faculty of Medicine, Ottawa, Canada

**Keywords:** Latent tuberculosis infection, Cascade of care, Arctic

## Abstract

**Background:**

A remote arctic region of Canada predominantly populated by Inuit with the country’s highest incidence of tuberculosis.

**Methods:**

The study was undertaken to describe the latent tuberculosis infection (LTBI) cascade of care and identify factors associated with non-initiation and non-completion of LTBI treatment. Data were extracted retrospectively from medical records for all patients with a tuberculin skin test (TST) implanted in Iqaluit, Nunavut between January 2012 and March 2016. Associations between demographic and clinical factors and both treatment non-initiation among and treatment non-completion were identified using log binomial regression models where convergence could be obtained and Poisson models with robust error variance where convergence was not obtained.

**Results:**

Of 2303 patients tested, 439 (19.1%) were diagnosed with LTBI. Treatment was offered to 328 patients, was initiated by 246 (75.0% of those offered) and was completed by 186 (75.6% of initiators). In multivariable analysis, older age (adjust risk ratio [aRR] 1.17 per 5-year increase, 95%CI:1.09–1.26) and undergoing TST due to employment screening (aRR 1.63, 95%CI:1.00–2.65, compared to following tuberculosis exposure) were associated with increased non-initiation of treatment. Older age (aRR 1.13, 95%CI: 1.03–1.17, per 5-year increase) was associated with increased non-completion of treatment.

**Conclusions:**

A similar rate of treatment initiation and higher rate of treatment completion were found compared to previous North American studies. Interventions targeting older individuals and those identified via employment screening may be considered to help to address the largest losses in the cascade of care.

## Background

Canada has a low overall incidence of tuberculosis (TB), yet the burden of the disease disproportionally falls on Indigenous populations and, in particular, on Inuit people. Inuit had the highest incidence of active TB in Canada in 2016 with a rate of 170.1 per 100,000 compared to 4.8 per 100,000 in Canada as a whole [1]. Nearly half of the Inuit population in Canada resides in the Territory of Nunavut with many others living in adjacent arctic regions collectively known as Inuit Nunangat (Inuit homeland) [2]. In an attempt to address the high burden of TB in these regions, the Government of Canada and Inuit Tapariit Kanatami (Inuit National Organization) have announced a goal to eliminate TB in Inuit Nunangat by 2030 [3]. Potential strategies to achieve this are being developed in a project led by Inuit [4].

Although analyses within arctic settings have yet to be performed, modelling studies assessing strategies for TB elimination globally and in the United States have been done [5, 6]. These studies concluded that widespread and effective treatment of latent tuberculosis infection (LTBI) is likely to be necessary for TB elimination [5, 6]. In order to design interventions to increase LTBI treatment rates, numerous steps between the decision to test for LTBI and completion of treatment (termed the LTBI cascade of care [7]) must be carefully examined and barriers overcome. Particular challenges in the Canadian arctic include the geographic isolation and challenging climate which limit access and available resources to the remote communities where many Inuit live [8]. Further, most healthcare professionals arrive from Southern Canada and are frequently unfamiliar with the local culture and language and there are difficulties in procurement and retention of experienced staff and prioritization among numerous competing health care priorities [8]. Finally, overcrowded housing and food insecurity are common [8].

Previous studies of LTBI in the Canadian arctic have focused on single interventions and none have examined the LTBI cascade of care under a routine program setting. In 2011, we conducted a TB awareness campaign followed by a door to door screening program to augment the local TB program in high-risk neighbourhoods in Iqaluit, Nunavut’s capital [9]. Among new LTBI cases identified, 24/31 (77%) who took at least one dose of directly observed twice weekly INH for nine months completed treatment (≥80% of doses within 1 year) [9]. In a recent study of a community screening program in Nunavik, Quebec, 85/120 (71%) newly identified or previously inadequately treated LTBI patients completed ≥80% of treatment doses (primarily self-administered rifampin for 4 months) [10].

The present study was undertaken to describe the LTBI cascade of care under regular program conditions in Iqaluit, Nunavut between January 2012 and March 2016 and to identify factors associated with non-initiation of LTBI treatment among patients offered treatment and non-completion of treatment among patients who initiated treatment.

## Methods

### Study design and population

Study data were collected retrospectively via review of electronic medical records and, where necessary, paper charts. Extracted data were entered into a Microsoft Excel (Microsoft Corporation, Redmond, WA, USA, 2017) database by a research nurse. Once the data were entered, a second research nurse reviewed the data to ensure completion and accuracy. Extracted data included demographic, clinical and treatment information for all patients in whom a tuberculin skin test (TST) was implanted in Iqaluit, Nunavut between January 2012 and March 2016*.* Key variables included patient age, ethnicity, TST result, TST indication, and whether treatment was offered, initiated and completed. Interferon gamma release assay (IGRA) results were not comprehensively collected but discordance between IGRA and TST results was captured when provided as a reason for not offering LTBI treatment.

### Study setting

Iqaluit is a remote community in the Canadian arctic and is the largest community in Inuit Nunangat. There are no roads linking Iqaluit to other communities. It is accessible by air year-round and by sea in the summer. In 2016, Iqaluit’s population was 7740, representing 21.5% of Nunavut’s population [2]. A majority (4265 people, 55.1%) of people in Iqaluit identify as Inuit [2]. The community reported 178 cases of active TB between 2010 and 2016 (36.4% of all cases in Nunavut during this time) with a mean of 26 cases per year (range 9–50) (unpublished data). Between 2007 and 2014 there were no new cases of HIV reported in Nunavut [11].

Health care services available in Iqaluit include public health (including LTBI testing and treatment and management of active TB) as well as primary and secondary care. Advanced medical interventions require medical transport to facilities in southern Canada. Health care interventions including diagnostic testing, nursing and physician assessment and TB medication are provided without charge.

All testing for LTBI in Iqaluit is performed via Iqaluit Public Health with TST being the recommended modality [12]. The IGRA is not used routinely but is performed in select cases at the discretion of the patient’s physician. TSTs are predominantly performed following exposure to active TB cases, following physician referral and as part of screening of school children in kindergarten and grade 6 and workers in some professions (e.g. healthcare workers) [12]. TSTs are strongly encouraged in these circumstances but not mandatory. Two step TSTs are performed when repeat testing is anticipated (e.g. healthcare workers) [12]. Public health nurses administer and read all tests as well as providing education regarding LTBI. TST interpretation was based upon the Canadian TB Standards which recommend a 10 mm cut off for test positivity in the absence of risk factors and a 5 mm cut off for those with recent TB contacts or other risk factors [13].

All patients with a positive TST are referred for physician assessment and treatment may be offered at the discretion of the assessing physician. The standard LTBI treatment regimen during the study period was 9 months of isoniazid given twice weekly via directly-observed therapy [12]. BCG vaccination is recommended for all infants in Nunavut before 12 months of age but is not mandatory [12]. Repeat BCG vaccination and vaccination at older ages is not performed [12]. In a previous study of residents of a high-risk neighbourhood of Iqaluit undergoing LTBI screening, 79% of participants for whom records were available had received BCG vaccination [14].

### Statistical analysis

We performed descriptive statistics on patient demographics and quantified the amount and reasons for loss to follow-up along each step in the LTBI cascade of care.

The primary analyses assessed the association between demographic and clinical factors and both treatment non-initiation among TST positive patients offered LTBI treatment and treatment non-completion among TST positive patients starting LTBI treatment. Patients could have had previous negative TSTs results but were included at the time of their first positive TST result. Clinical factors included the reason for performing TST. When TST was performed as part of a contact tracing investigation, the reason for TST was termed “TB exposure”. Treatment non-initiation was defined as failure to receive at least one dose of a drug for LTBI. Treatment non-completion was defined as taking < 80% of prescribed doses within 12 months of treatment initiation. There is no universally accepted definition of treatment completion for LTBI regimens and widely variable definitions have been used in previous studies [15]. Given this, sensitivity analyses were performed in which treatment non-completion was defined as taking < 90% of doses within 12 months and as taking < 100% of doses within 12 months.

Unadjusted risk ratios (RRs) for each demographic and clinical factor were estimated using univariable regression models while adjusted RRs (aRRs) were estimated using multivariable models. Log binomial models were used for all analyses except for those examining treatment non-initiation. In this instance, log binomial models failed to converge therefore Poisson models with robust error variance [16] were used. Key variables were included in multivariable models based on their clinical importance while additional variables were assessed for inclusion based on descriptive and univariate analyses as well as model fit statistics (see Additional file 1: Appendix 1).

Two secondary analyses were performed. The first examined factors associated with TST positivity among those for whom a TST result was obtained and the second evaluated factors associated with failure to obtain a TST result among all patients in whom a TST was implanted. Risk ratios in both of these analyses were estimated using log binomial regression models fit using generalized estimating equations. All TST results were included in the analysis and an exchangeable correlation matrix was used to adjust for correlation between the results of multiple TSTs performed on the same individual.

For regression models in which all variables had < 10% missing data, a complete case analysis was performed. This cut off was selected a priori with the assistance of a biostatistician. When ≥10% of values were missing for at least one variable, all missing values were imputed using logistic regression with the imputation model variables including sex, age, indication for TST, year of TST and ethnicity. Twenty datasets were imputed using the SAS PROC MI procedure and regression models were fit for each. Estimates of coefficients and variance from these models were then combined according to Rubin’s Rules as implemented in the SAS PROC MIANALYZE procedure. Complete case analyses were performed for comparison.

Statistical analysis was carried out using SAS software, version 9.4 (SAS Institute Inc., Cary, NC, USA, 2017).

## Results

### Demographic characteristics

Characteristics of the 2303 patients in whom TSTs were implanted during the study period are presented in Table 1. The mean age at first TST was 25.3 years (standard deviation 16.3, range 0–86). The ethnicity of 425 patients (18.5%) was unknown while 1220 (53.0%) were Inuit and 658 (28.6%) were non-Inuit. There were 619 patients (26.9%) with more than one TST. The most common reason for repeat TST was TB exposure (441/870 tests, 50.7%) followed by self or physician referral (182/870 tests, 20.9%), employment screening (175/870 tests, 20.1%) and school screening (30/870 tests, 3.4%). There were 42 repeat tests performed for unknown or other reasons (4.8%).

**Table 1:** Demographic characteristics of included patients (*n* = 2303 individuals with 3173 tuberculin skin tests). TST = tuberculin skin test, SD = standard deviation

	All patients (*n* = 2303)	Diagnosed with latent tuberculosis infection (*n* = 439)	Diagnosed with active tuberculosis (*n* = 17)	Not diagnosed with latent or active tuberculosis (*n* = 1847)
Characteristic				
Age at first TST, mean (SD)				
Males	25.2 (17.1)	25.1 (17.7)	30.6 (18.7)	25.1 (16.9)
Females	25.4 (15.6)	24.4 (16.5)	29.1 (19.5)	25.6 (15.3)
Total	25.3 (16.3)	24.8 (17.1)	29.9 (18.5)	25.4 (16.0)
Age at first TST, n (%)				
< 18 years	784 (34.0)	169 (38.5)	4 (23.5)	611 (33.1)
18–35 years	919 (39.9)	157 (35.8)	4 (23.5)	758 (41.0)
> 35 years	600 (26.1)	113 (25.8)	9 (52.9)	478 (25.9)
Sex, n (%)				
Male	1065 (46.2)	224 (51.0)	9 (52.9)	832 (45.0)
Female	1238 (53.8)	215 (49.0)	8 (47.1)	1015 (55.0)
Ethnicity, n (%)				
Inuit	1220 (53.0)	290 (66.1)	16 (94.1)	914 (49.5)
Non-Inuit	658 (28.6)	90 (20.5)	0 (0)	568 (30.8)
Unknown	425 (18.5)	59 (13.4)	1 (5.9)	365 (19.8)
Number of TSTs performed, n (%)				
1	1684 (73.1)	344 (78.4)	16 (94.1)	1324 (71.7)
2	441 (19.1)	74 (16.9)	1 (5.9)	366 (19.8)
3	128 (5.6)	17 (3.9)	0 (0)	111 (6.0)
> 3	50 (2.2)	4 (0.9)	0 (0)	46 (2.5)
TST Result, n (%)				
Ever positive	456 (19.8)	439 (100)	17 (100)	0 (0)
Never positive	1847 (80.2)	0 (0)	0 (0)	1847 (100)

### LTBI Cascade of care

**Fig. 1:**
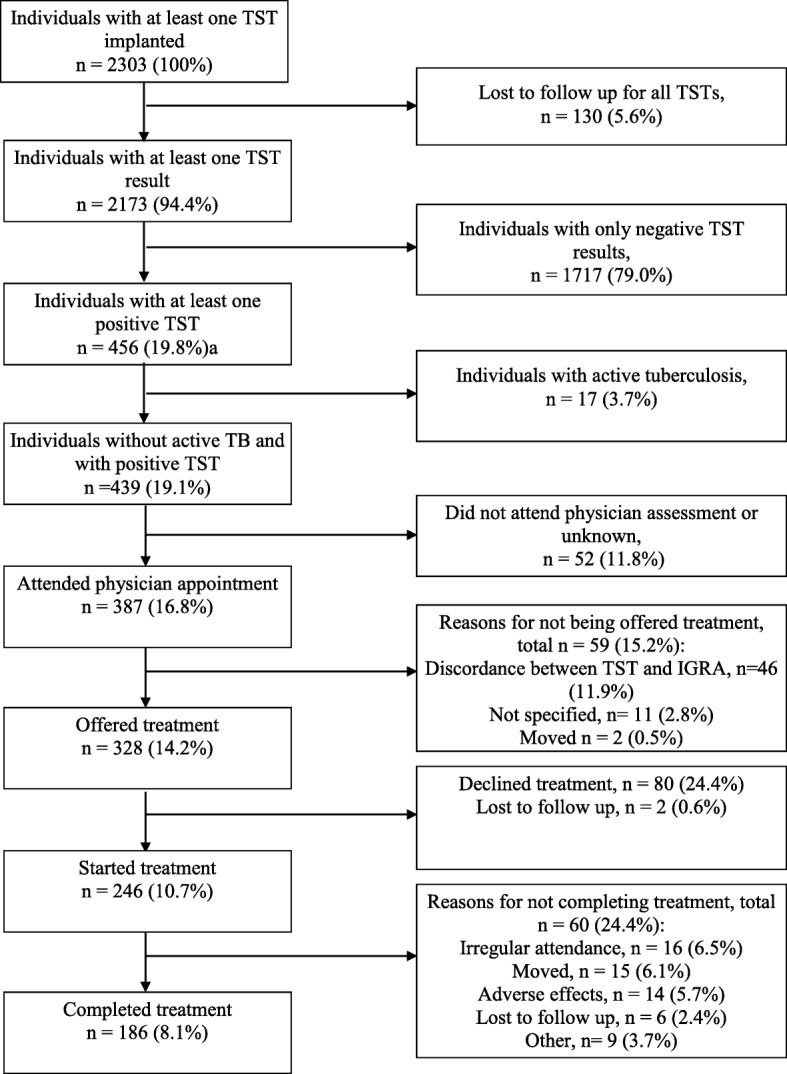
Latent tuberculosis cascade of care in Iqaluit, Nunavut for January 2012–March 2016. TST = tuberculin skin test; IGRA = interferon gamma release assay. Percentages in the left-hand column represent the percentage of patients remaining in the cascade compared to the total number of patients who underwent at least one TST. Percentages in the right-hand column represent the percentage of patients exiting the cascade compared to the total number of patients remaining at that point in the cascade

The LTBI cascade of care for the study period is presented in Fig. 1. A total of 3173 TSTs were performed with a median of 1 test per patient (range 1–8). Of the 2303 patients with at least one implanted TST, no TST result was obtained for 130 (5.6%). Overall, 462 TSTs (14.6%) performed in 456 patients were positive. The 456 patients with a positive TST result represent 19.8% of all patients tested. Of these 456 patients, 17 were subsequently determined to have active tuberculosis. Thus, 439 patients (19.1% of screened patients) were considered to have LTBI and referred for consideration of treatment. Thus, 5.2 patients were screened for each LTBI case identified.

Physician appointments were attended by 387 (88.2%) of the 439 patients referred and 328 (84.8% of assessed patients) were offered treatment. The most common reason for not offering treatment was discordance between TST and IGRA (46 of 59 patients not offered treatment). Treatment was started by 246 patients which is 75.0% of the 328 patients offered treatment and 56.0% of the 439 patients with LTBI. Treatment was completed by 186 (75.6%) of the 246 patients who initiated treatment. The most common reasons provided for not completing treatment were irregular attendance (16 of 60 non-completers), moving (15 of 60 non-completers) and adverse effects of treatment (14 of 60 non-completers). When defining completion as receipt of 90% or 100% of doses within 12 months, 175 patients (71.1% of those initiating treatment) and 150 patients (60.9% of those initiating treatment) completed treatment, respectively. Isoniazid for 9 months was the treatment regimen for 232 (94.3%) of patients starting therapy while 6 patients (2.4%) were treated with 4 months of rifampin and 8 (3.3%) with unknown or other regimens. The number of patients screened for each patient who completed treatment was 12.4.

### Primary analysis – factors associated with non-initiation and non-completion of treatment

Table 2 describes associations between clinical and demographic factors and non-initiation of LTBI treatment. In unadjusted analyses, increased age (RR 1.17 per 5-year increase, 95% confidence interval 1.13–1.25), non-Inuit ethnicity (RR 2.92, 1.97–4.32) and undergoing TST due to employment screening (RR 2.13,1.34–3.39, compared to following TB exposure) were associated with increased non-initiation of treatment. However, in the adjusted analysis, only increased age (aRR 1.17 per 5-year increase, 1.09–1.26) and undergoing TST due to employment screening (aRR 1.63, 1.00–2.65, compared to following TB exposure) were associated with increased non-initiation of treatment.

**Table 2:** Risk ratios for non-initiation of treatment by demographic and clinical characteristic among patient offered latent tuberculosis infection treatment. Risk ratios marked with an asterisk (*) are statistically significant. CI = confidence interval

Potential Risk factor	Non-initiators/category total (%)	Unadjusted risk ratio (95% CI)	Adjusted risk ratio^b^ (95% CI), *n* = 280 patients^a^
Age, years (per 5-year increase)	–	1.19 (1.13–1.25)*	1.17 (1.09–1.26)*
Sex			
Male	37/163 (22.7%)	Reference	Reference
Female	43/163 (26.4%)	1.16 (0.79–1.70)	1.08 (0.71–1.65)
Ethnicity			
Inuit	40/242 (16.5%)	Reference	Reference
Non-Inuit	27/56 (48.2%)	2.92 (1.97–4.32)*	1.52 (0.91–2.54)
Indication for TST			
Tuberculosis exposure	34/173 (19.7%)	Reference	Reference
Employment screening	18/43 (41.9%)	2.13 (1.34–3.39)*	1.63 (1.00–2.65)*
School screening	1/14 (7.1%)	0.36 (0.05–2.31)	1.11 (0.16–7.58)
Self or physician referral	22/76 (28.9%)	1.47 (0.93–1.14)	0.99 (0.56–1.74)
Year of assessment			
2012	28/129 (21.7%)	Reference	–
2013	12/72 (16.7%)	0.78 (0.42–1.43)	–
2014	23/71 (32.4%)	1.47 (0.92–2.36)	–
2015	14/37 (37.8%)	1.74 (0.99–2.97)	–
2016	3/12 (25.0%)	1.15 (0.41–3.23)	–

^a^Among the 328 patients offered treatment, complete data were available for 280. Data were missing regarding ethnicity in 29 (8.8%), year of assessment in 5 (1.5%), indication for TST in 20 (6.1%) and treatment initiation in 2 (0.6%)

^b^Model included age, sex, ethnicity and indication for TST

Table 3 lists risk ratios for non-completion of LTBI treatment among those who started treatment. In both unadjusted and adjusted analyses, increased age (RR 1.10, 1.03–1.17; aRR 1.13, 1.03–1.17, per 5-year increase) was associated with increased non-completion of treatment. This association remained in a sensitivity analysis defining treatment non-completion as receipt of < 90% of treatment doses, (aRR 1.08, 1.01–1.15). However, when defining treatment non-completion as receipt of < 100% of treatment doses, this association was not statistically significant (aRR 1.04, 0.98–1.10).

**Table 3:** Risk ratios for non-completion of treatment among patients starting treatment for latent tuberculosis infection. Risk ratios marked with an asterisk (*) are statistically significant. CI = confidence interval

Potential Risk factor	Non-completers/ category total (%)	Unadjusted risk ratio (95% CI)	Adjusted risk ratio^b^ (95% CI), *n* = 208 patients^a^
Age, years (per 5-year increase)	–	1.10 (1.03–1.17)*	1.13 (1.04–1.22)*
Sex			
Male	25/124 (20.2%)	Reference	Reference
Female	23/110 (20.9%)	1.04 (0.63–1.72)	1.19 (0.71–1.99)
Ethnicity			
Inuit	39/193 (20.2%)	Reference	Reference
Non-Inuit	8/27 (29.6%)	1.47 (0.77–2.80)	0.84 (0.41–1.73)
Indication for TST			
Tuberculosis exposure	29/133 (21.8%)	Reference	Reference
Employment screening	8/23 (34.8%)	1.60 (0.84–3.04)	1.44 (0.75–2.75)
School screening	1/14 (7.1%)	0.33 (0.05–2.23)	0.68 (0.10–4.57)
Self or physician referral	9/51 (17.6%)	0.81 (0.41–1.59)	0.73 (0.38–1.43)
Year of assessment			
2012	20/98 (20.4%)	Reference	–
2013	7/46 (15.2%)	0.75 (0.34–1.64)	–
2014	12/56 (21.4%)	1.05 (0.56–1.98)	–
2015	6/21 (28.6%)	1.40 (0.64–3.06)	–
2016	2/9 (22.2%)	1.09 (0.30–3.93)	–

^a^Among the 246 patients who started treatment, complete data were available for 208. Data were missing regarding ethnicity in 15 (6.1%), year of assessment in 16 (6.5%), indication for TST in 14 (5.7%) and treatment completion in 12 (3.7%)

^b^Model included age, sex, ethnicity and indication for TST

### Secondary analyses - TST positivity and failure to obtain a result

Factors potentially associated with TST positivity are presented in Table 4. A reduced risk of having a positive result was associated with female sex (aRR 0.80, 0.68–0.95), non-Inuit ethnicity (aRR 0.68, 0.54–0.86) and obtaining a TST due to employment screening (aRR 0.58, 0.45–0.75), school screening (aRR 0.48, 0.33–0.70) or physician or self-referral (aRR 0.69, 0.57–0.83). TSTs performed in 2013, 2014 and 2015 were less likely to be positive than those performed in 2012 (Table 4).

**Table 4:** Risk ratios for TST positivity by demographic and clinical characteristic. Adjusted results are presented for complete case analysis (*n* = 1878 patients^a^) and analysis using combined results of 20 imputed datasets (*n* = 2303 patients). Risk ratios marked with an asterisk (*) are statistically significant. CI = confidence interval

Potential Risk factor	Patients with positive TST/ category total (%)	Risk ratio from univariate models^b^ (95% CI)	Adjusted risk ratio^c^ (95% CI), complete case	Adjusted risk ratio^c^ (95% CI), multiple imputation
Age, years (per 5-year increase)	–	0.99 (0.97–1.02)	1.00 (0.97–1.04)	1.01 (0.98–1.04)
Sex				
Male	233/1065 (21.9%)	Reference	Reference	Reference
Female	223/138 (18.0%)	0.80 (0.68–0.95)*	0.78 (0.65–0.94)*	0.80 (0.68–0.95)*
Ethnicity				
Inuit	306/1220 (25.1%)	Reference	Reference	Reference
Non-Inuit	90/658 (13.7%)	0.58 (0.47–0.73)*	0.64 (0.50–0.83)*	0.68 (0.54–0.86)*
Indication for TST				
Tuberculosis exposure	199/606 (32.8%)	Reference	Reference	Reference
Employment screening	67/506 (13.2%)	0.45 (0.35–0.57)*	0.59 (0.45–0.78)*	0.58 (0.45–0.75)*
School screening	34/297 (11.4%)	0.40 (0.28–0.57)*	0.36 (0.22–0.60)*	0.48 (0.33–0.70)*
Self or physician referral	124/746 (16.6%)	0.62 (0.51–0.75)*	0.67 (0.55–0.83)*	0.69 (0.57–0.83)*
Year of TST				
2012	201/759 (26.5%)	Reference	Reference	Reference
2013	101/547 (18.5%)	0.64 (0.52–0.80)*	0.64 (0.51–0.80)*	0.65 (0.53–0.80)*
2014	95/498 (19.1%)	0.75 (0.61–0.92)*	0.73 (0.59–0.92)*	0.77 (0.63–0.94)*
2015	45/425 (10.6%)	0.39 (0.30–0.51)*	0.41 (0.30–0.56)*	0.45 (0.34–0.59)*
2016	12/74 (16.2%)	0.66 (0.42–1.05)	0.75 (0.45–1.24)	0.77 (0.50–1.20)

^a^Data regarding ethnicity were missing for 472 TSTs (14.9%) in 425 patients (18.5%) and were estimated using multiple imputation. Data on indication were missing for 189 TSTs (6.0%) in 148 patients (6.4%)

^b^Univariate models adjusted for the correlation between multiple TSTs performed on the same individual using an exchangeable correlation matrix

^c^Model included age, sex, ethnicity, indication for TST and year of TST and adjusted for multiple TSTs performed on the same individual using an exchangeable correlation matrix

Associations with failure to obtain a TST result are presented in Table 5. Having a TST planted in 2015 (aRR1.52, 1.04–2.22, compared to 2012) and obtaining a TST due to physician or self-referral (aRR 1.64, 1.17–2.30, compared to following TB exposure) were associated with an increased risk of failure to obtain a TST result.

**Table 5:** Risk ratios for failure to obtain a TST result by demographic and clinical characteristic. Results are presented for complete case analysis (*n* = 1878 patients^a^) and analysis using combined results of 20 imputed datasets (*n* = 2303 patients). RRs marked with an asterisk (*) are statistically significant. CI = confidence interval

Potential Risk factor	Patients with no TST results/ category total (%)	Risk ratio from univariate models^b^ (95% CI)	Adjusted risk ratio^c^ (95% CI), complete case	Adjusted risk ratio^c^ (95% CI), multiple imputation
Age, years (per 5-year increase)	–	0.98 (0.95–1.02)	–	–
Sex				
Male	69/1065 (6.5%)	Reference	–	–
Female	61/1238 (4.9%)	0.87 (0.67–1.13)	–	–
Ethnicity				
Inuit	48/1220 (3.9%)	Reference	Reference	Reference
Non-Inuit	28/658 (4.3%)	0.76 (0.54–1.06)	0.68 (0.47–0.98)*	0.79 (0.58–1.06)
Indication for TST				
Tuberculosis exposure	20/606 (3.3%)	Reference	Reference	Reference
Employment screening	26/506 (5.1%)	1.22 (0.82–1.83)	1.08 (0.65–1.78)	1.16 (0.77–1.76)
School screening	25/297 (8.4%)	1.52 (0.95–2.42)	1.26 (0.68–2.29)	1.32 (0.83–2.11)
Self or physician referral	45/746 (6.0%)	1.72 (1.22–2.42)*	1.65 (1.12–2.44)*	1.64 (1.17–2.30)*
Year of TST				
2012	28/759 (3.7%)	Reference	Reference	Reference
2013	29/547 (5.3%)	1.27 (0.88–1.83)	1.38 (0.88–2.16)	1.25 (0.87–1.81)
2014	31/498 (6.2%)	1.18 (0.80–1.74)	1.08 (0.65–1.79)	1.16 (0.79–1.71)
2015	36/425 (8.5%)	1.52 (1.05–2.19)*	1.19 (0.70–2.02)	1.52 (1.04–2.22)*
2016	6/74 (8.1%)	1.76 (0.93–3.34)	1.98 (0.94–4.15)	1.71 (0.91–3.21)

^a^Data regarding ethnicity were missing for 472 TSTs (14.9%) in 425 patients (18.5%) and were estimated using multiple imputation. Data on indication were missing for 189 TSTs (6.0%) in 148 patients (6.4%)

^b^Univariate models adjusted for the correlation between multiple TSTs performed on the same individual using an exchangeable correlation matrix

^c^Model included ethnicity, indication for TST and year of TST and adjusted for the correlation between multiple TSTs performed on the same individual using an exchangeable correlation matrix

## Discussion

This retrospective cohort study, 19.1% of referred patients were diagnosed with LTBI during a 51-month period in a routine screening program in a remote Canadian arctic region with a predominantly Inuit population. The cascade of care demonstrated high rates of LTBI treatment initiation (75%) and completion (76%). Older age and receiving a TST during employment screening were associated with non-initiation of treatment while only older age was associated with non-completion of treatment.

Despite the challenges of delivering care in a remote setting, the rate of treatment initiation in the present study was broadly similar to those from large North American studies while the rate of treatment completion was higher. In a 2003 study of over 13,000 LTBI cases across 29 American jurisdictions, 69.9% of all LTBI patients took at least one treatment dose while 63.7% of these patients completed ≥80% of prescribed doses [17]. In a 2010 study involving 32 clinics, 82.9% of LTBI patients offered treatment initiated it and 47.3% of initiators completed 100% of treatment doses within one year [18]. Finally, only 31.3% of patients prescribed LTBI treatment filled prescriptions for all doses in a 2013 analysis of health administrative data in Quebec [19]. The higher rates of treatment completion in Iqaluit may be related to the use of directly observed therapy and a twice weekly regimen which requires fewer total doses than traditional daily therapy.

In our study, non-initiation of treatment among patients for whom treatment was recommended represented the largest source of loss of TST positive patients from the cascade of care. The increased risk of non-initiation among older patients and those identified via employment screening may reflect a less favourable balance of treatment risk and benefit among these groups. The risk of adverse effects, particularly hepatotoxicity, due to isoniazid increases with age [20, 21] which may have led to reluctance among older patients to start treatment. Further, LTBI reactivation risk is highest within the first 2 years following infection [22]. Thus, those with positive testing in screening programs but with unknown or remote TB exposure may anticipate less benefit from treatment than those with a known, recent exposure. Of note, the Canadian TB standards do not include an age-based cut off above which LTBI treatment is not recommended but, instead, recommend weighing the risks and benefits of treatment on an individual basis [13].

To improve initiation rates, interventions targeting older individuals and those identified via employment screening should be considered. This could include the expanded use of less hepatotoxic regimens such as the 12- week regimen of rifapentine and isoniazid (3HP) [23] which could be particular beneficial for older patients. Further, providing videos of community elders discussing the nature and benefits of LTBI treatment to older patients may help to assist in establishing understanding and openness to considering such therapies potentially increasing initiation. Finally, providing education regarding the importance of LTBI testing and treatment to employees starting jobs which will require LTBI screening. This may help to shape opinions on these topics early, potentially increasing treatment initiation later when LTBI is diagnosed.

Non-completion of treatment represented the second largest loss of TST positive patients from the cascade of care and was associated with older age. As noted above, adverse effects related to isoniazid are more common among older patients [20, 21] and may have contributed to reduced completion in this group. The use of 3HP has been associated with both increased completion rates and reduced hepatotoxicity compared to isoniazid alone [15, 23]. As such, this alternative regimen may be of particular benefit in this setting with the potential to increase both initiation and completion.

Irregular appointment attendance was the most commonly listed reason for non-completion. The use of shorter treatment regimens has been associated with increased completion [15]. In the present study, the vast of majority of patients (94.3%) used a 9-month regimen. Thus, a change to a shorter regimen, which could include the 3HP regimen mentioned above, may improve appointment attendance by reducing the number of required clinic visits.

Physicians frequently cited discordance between TST and IGRA results as a reason for not offering LTBI treatment. The use of IGRA as a confirmatory test following TST is not endorsed by Canadian or World Health Organization guidelines [13, 24]. However, this strategy is recommended for some low risk patients in American guidelines [25] and a recent trial found it to be non-inferior to TST alone [26], suggesting that it may be reasonable in some cases.

A moderate number of patients were lost to follow up before obtaining a TST result and many patients with positive TSTs did not attend physician appointments. Perhaps standardized videos emphasizing TB prevention presented by Inuit community members to Inuit patients could improve attendance.

By including the results of all TSTs performed in Iqaluit over the study period, our study provides a comprehensive overview of LTBI identification and management in the largest community in Canada’s highest TB incidence region. This represents the first comprehensive data published from the Inuit Nunangat. A further strength is that all LTBI treatment in Iqaluit is directly-observed, minimizing misclassification error.

However, the study is limited by the relatively small number of variables on which data were obtained. This is in part related to this study’s retrospective design which meant that only data listed in medical records could be extracted. As a result, the potential impact of important clinical and socioeconomic factors could neither be assessed nor adjusted for. Additionally, post-hoc statistical analysis regarding the listed reasons for treatment non-completion was considered but rejected because numbers in each category were low (*n* ≤ 16 for all categories), leading to very limited statistical power. This prevented a more detailed analysis of potential causes of non-completion. While power could have been increased by increasing sample size, study dates were limited by the availability of data.

Given these limitations, richer detail may be better obtained in future through qualitative studies exploring patients’ reasons for not initiating or completing treatment. The common themes could then provide targets for future interventions to improve treatment initiation and completion.

A further limitation is that IGRA results were not comprehensively captured. Although it would not have been in keeping with recommended local practices [12], it is possible that some patients were tested exclusively with IGRA and thus not included in our study.

An additional limitation is that it is possible that some patients underwent LTBI treatment prior to the study but were included following a repeat TST. This could have led to an underestimation of the treatment completion rate. However, we feel that this would not be not common. This is because it is not standard practice in Iqaluit to treat patients unless they have a positive TST (no patient with a negative TST received LTBI therapy during the study period) and nearly all of those with repeat testing initially had negative TSTs (only 6 patients with a positive TST had a repeat test). Since TSTs relatively infrequently revert to negative after treatment [27], it is unlikely that many of those with repeat TSTs had undergone a previous course of treatment.

## Conclusions

In our analysis of a routine TB program in the largest community of a remote Canadian arctic region over a 51-month period, 19.1% of referred patients were diagnosed with LTBI. Despite the challenges of delivering care in this setting, a similar rate of treatment initiation and higher rate of treatment completion were found compared to previous North American studies. Interventions targeting older individuals and those identified via employment screening may be considered to help to address the largest losses seen in the cascade of care.

## Supplementary information

**Additional file 1.** Supplementary Appendix. This includes expanded study definitions, additional details regarding the model building strategy and detailed results of sensitivity analyses.

## Data Availability

The data that support the findings of this study are available on reasonable request from the corresponding author. The data are not publicly available because they contain information that could compromise research participant privacy/consent.

## Abbreviations

aRR: adjusted risk ratio
IGRA: Interferon gamma release assay
LTBI: Latent tuberculosis infection
RR: Risk ratio
TB: Tuberculosis
TST: Tuberculin skin test
