# Supplementary material for: The latent tuberculosis infection cascade of care in Iqaluit, Nunavut, 2012–2016
Source: BMC Infect Dis. 2019 Oct 24;19:890. doi: 10.1186/s12879-019-4557-3 (PMC6814024; doi:10.1186/s12879-019-4557-3)
Supplement: Supplementary file 1 — Additional file 1. Supplementary Appendix. This includes expanded study definitions, additional details regarding the model building strategy and detailed results of sensitivity analyses. [file 12879_2019_4557_MOESM1_ESM.docx]

**Supplementary Appendix**

The latent tuberculosis infection cascade of care in Iqaluit, Nunavut, 2012-2016

Christopher Pease^1,2^ (cpease@toh.ca), Alice Zwerling^2^ (azwerlin@uottawa.ca), Ranjeeta Mallick^3^ (rmallick@ohri.ca), Mike Patterson^5^ (mpatterson@gov.nu.ca), Patricia Demaio^5^ (pdemaio@gov.nu.ca), Sandy Finn^5^ (sfinn@gov.nu.ca), Jean Allen^6^ (jallen@tunngavik.com), Deborah Van Dyk^3^ (dvandyk@ohri.ca), Gonzalo G. Alvarez^1,2,3,4^ (galvarez@toh.ca)

1=Ottawa Hospital, Department of Medicine, Ottawa, Canada

2= Ottawa University School of Epidemiology and Public Health, Ottawa, Canada

3=Ottawa Hospital Research Institute, Ottawa, Canada

4=Ottawa University Faculty of Medicine, Ottawa, Canada

5=Ministry of Health, Government of Nunavut, Iqaluit, Nunavut

6=Nunavut Tunngavik Inc, Iqaluit, Nunavut

**Appendix 1. Supplementary Methods**

***Variable Definitions***

Tuberculin skin tests (TSTs) were defined as positive based on the assessment of Iqaluit Public Health. Territorial guidance during the study period stated that TSTs should be interpreted based on the Canadian Tuberculosis Standards, 5^th^ edition(1). Briefly, these standards recommend that a TST be considered positive if the induration size is ≥5mm in those with a close infectious contact, fibronodular disease on chest x-ray or HIV; and ≥10mm in all others(2).

Treatment non-initiation was defined failure to receive at least one dose of a drug for LTBI. Treatment non-completion was defined as taking <80% of prescribed doses within 12 months of treatment initiation.

***Model building***

The general approach to building regression models involved a mixed approach with the inclusion of certain variables based on their clinical importance and additional variables being included or excluded based on the results of descriptive and univariate analyses as well as model fit statistics.

*Primary Analysis*

For the primary analyses, the indication for performing TST was included in final models because treatment indication has been a strong predictor of treatment initiation (3,4) and treatment completion(3) in other populations. Inuit ethnicity was included because uniquely high rates of TB among this group (5) make it of a priori interest. Further, age was included because the risk of adverse effects of isoniazid increases which age(6,7). It was thus suspected that older patients may be less likely to compete therapy for this reason making it a potential confounder if also related to other variables in the analysis. Although data are less robust for LTBI, important differences between sexes in the burden and treatment active TB disease have long been recognized(8,9). For this reason, sex was included in the final models.

Year of physician visit was not included in the final treatment non-initiation or non-completion regression model based on examination of study data. Specifically, to evaluate a potential temporal effect, the year of physician visit was compared to treatment initiation and completion graphically. No clear temporal trend was noted (data not shown). The potential impact of this variable was further evaluated in univariate analysis and no association was found for either treatment initiation or completion (95% confidence intervals of risk ratios (RRs) included 1). Finally, model fit statistics did not substantially improve with inclusion in the model. For the model of treatment initiation, the quasi-likelihood under the independence model criterion (QIC) was QIC 410.45 in the model including year of physician visit and QIC 404.38 without it (smaller numbers indicating better fit). For the completion model, the Akaike’s Information Criterion (AIC) was 303.42 with inclusion and 302.87 without (smaller numbers indicate better fit) and the Bayesian information criterion (BIC) was 340.45 with inclusion and 326.57 without (smaller numbers indicate better fit). As such, this variable was not included in the final model.

*Secondary Analyses*

For secondary analyses, a similar approach was used. For the analysis of risk factors for TST positivity, previous analyses of high risk neighbourhoods in Iqaluit have shown age(10) and ethnicity (insert SDH citation) to be associated with LTBI and thus these variables were included in the model. Indication for TST and sex were retained based on significant associations in univariate analyses (95% confidence intervals of RRs not including 1) and because of improved model fit with the variables. Specifically, the QIC was substantively increased in the model without these variables than in those with them included, indicating worse fit (QIC 1977.64 with both variables vs 2143.25 without indication for TST and 1982.62 without sex). Year of TST was included based on graphical analysis suggesting a possible trend (data not shown), statistical significance of univariate analyses (95% confidence intervals of RRs not including 1) and worsened model fit without the variable (QIC 1977.64 with the variable vs 2013.87 without).

For the analysis of factors associated with failure to obtain a TST result, indication for testing was included based on a previous meta-analysis showing an association between test indication and completion of latent tuberculosis infection testing(3). Ethnicity was included both because of the high rates of active TB in the population and because high levels of adverse socioeconomic indicators among Inuit people (11,12) was felt to put them at higher risk of loss to follow up. Year of TST was included based on a possible trend in graphical analysis and statistical significance in the risk of failure to return for TST reading in univariate analysis between tests performed in 2012 vs 2015 (95% confidence intervals of RR not including 1) although model fit was similar with and without the variable (QIC 1111.02 with the variable and 1110.97 without). Sex and age were not included because univariate analysis showed no significant associations with the outcome (95% confidence intervals of RR not including 1) and model fit worsened with its inclusion (QIC 1111.015 with sex and age vs 1108.97 without sex and 1109.39 without age).

**Appendix 2. Results of sensitivity analyses.**

Table S1 shows the results of regression models examining associations between clinical and demographic factors and non-completion of treatment defined as receipt of <90% of prescribed doses of therapy within 12 months of the first dose. Table S2 shows similar results when defining treatment non-completion as receipt of <100% of prescribed doses of therapy within 12 months of the first dose.

**Table S1**. Results of a sensitivity analysis defining treatment non-completion as receipt of <90% of prescribed doses of therapy within 12 months of the first dose. Risk ratios (RRs) by demographic and clinical characteristic for non-completion of treatment among patients starting treatment for latent tuberculosis infection in Iqaluit, Nunavut between January 2012 and March 2016. RRs marked with an asterisk (*) are statistically significant. CI = confidence interval. n = 208 patients^1^.

| Potential Risk factor | Unadjusted risk ratio (95% CI) | Adjusted risk ratio (95% CI) |
| --- | --- | --- |
|  |  |  |
| Age, years (per 5-year increase) | 1.05 (1.00-1.11)* | 1.08 (1.01-1.15)* |
|  |  |  |
| Sex |  |  |
| Male | Reference | Reference |
| Female | 1.35 (0.91-2.01) | 1.49 (0.98-2.28) |
|  |  |  |
| Ethnicity |  |  |
| Inuit | Reference | Reference |
| Non-Inuit | 1.18 (0.68-2.04) | 0.90 (0.50-1.65) |
|  |  |  |
| Indication for TST |  |  |
| TB exposure | Reference | Reference |
| Employment screening | 1.32 (0.77-2.28) | 1.19 (0.69-2.06) |
| School screening | 0.24 (0.04-1.59) | 0.40 (0.06-2.62) |
| Self or physician referral | 0.86 (0.51-1.44) | 0.83 (0.49-1.41) |
|  |  |  |
| Year of assessment |  |  |
| 2012 | Reference | - |
| 2013 | 1.00 (0.81-1.22) | - |
| 2014 | 1.06 (0.87-1.30) | - |
| 2015 | 0.85 (0.60-1.21) | - |
| 2016 | 1.09 (0.75-1.58) | - |

^1^Among the 246 patients who started treatment, complete data were available for 207. Data were missing regarding ethnicity in 15 (6.1%), indication for TST in 14 (5.7%) and treatment completion in 12 (3.7%).

**Table S2.** Results of a sensitivity analysis defining treatment non-completion as receipt of <100% of prescribed doses of therapy within 12 months of the first dose. Risk ratios (RRs) by demographic and clinical characteristic for non-completion of treatment among patients starting treatment for latent tuberculosis infection in Iqaluit, Nunavut between January 2012 and March 2016. RRs marked with an asterisk (*) are statistically significant. CI = confidence interval. n = 208 patients^1^.

| Potential Risk factor | Unadjusted risk ratio (95% CI) | Adjusted risk ratio (95% CI) |
| --- | --- | --- |
|  |  |  |
| Age, years (per 5-year increase) | 1.03 (0.99-1.08) | 1.04 (0.98-1.10) |
|  |  |  |
| Sex |  |  |
| Male | Reference | Reference |
| Female | 1.24 (0.91-1.70) | 1.25 (0.90-1.74) |
|  |  |  |
| Ethnicity |  |  |
| Inuit | Reference | Reference |
| Non-Inuit | 1.50 (0.78-2.86) | 0.76 (0.45-1.30) |
|  |  |  |
| Indication for TST |  |  |
| TB exposure | Reference | Reference |
| Employment screening | 1.24 (0.78-1.95) | 1.29 (0.81-2.05) |
| School screening | 0.55 (0.20-1.54) | 0.86 (0.33-2.27) |
| Self or physician referral | 1.10 (0.75-1.59) | 0.88 (0.53-1.46) |
|  |  |  |
| Year of assessment |  |  |
| 2012 | Reference | - |
| 2013 | 1.01 (0.78-1.31) | - |
| 2014 | 1.08 (0.84-1.40) | - |
| 2015 | 0.86 (0.57-1.32) | - |
| 2016 | 1.10 (0.68-1.80) | - |

^1^Among the 246 patients who started treatment, complete data were available for 207. Data were missing regarding ethnicity in 15 (6.1%), indication for TST in 14 (5.7%) and treatment completion in 12 (3.7%).

**References**

1. Nunavut TB Control and Elimination Manual. Government of Nunavut.; 2010.

2. Long R. Canadian Tuberculosis Standards, 5th Edition [Internet]. 2007. Available from: http://publications.gc.ca/collections/Collection/H49-146-2000E.pdf

3. Alsdurf H, Hill PC, Matteelli A, Getahun H, Menzies D. The cascade of care in diagnosis and treatment of latent tuberculosis infection: a systematic review and meta-analysis. The Lancet Infectious Diseases. 2016 Nov;16(11):1269–78.

4. Horsburgh CR, Goldberg S, Bethel J, Chen S, Colson PW, Hirsch-Moverman Y, et al. Latent TB infection treatment acceptance and completion in the United States and Canada. Chest. 2010 Feb;137(2):401–9.

5. Vachon J, Gallant V, Siu W. Tuberculosis in Canada, 2016. Canada Communicable Disease Report. 2018;44(3/4):75–81.

6. Kunst H, Khan KS. Age-related risk of hepatotoxicity in the treatment of latent tuberculosis infection: a systematic review. Int J Tuberc Lung Dis. 2010 Nov;14(11):1374–81.

7. Hosford JD, von Fricken ME, Lauzardo M, Chang M, Dai Y, Lyon JA, et al. Hepatotoxicity from antituberculous therapy in the elderly: a systematic review. Tuberculosis (Edinb). 2015 Mar;95(2):112–22.

8. Horton KC, MacPherson P, Houben RMGJ, White RG, Corbett EL. Sex Differences in Tuberculosis Burden and Notifications in Low- and Middle-Income Countries: A Systematic Review and Meta-analysis. PLoS Med. 2016 Sep;13(9):e1002119.

9. Diwan VK, Thorson A. Sex, gender, and tuberculosis. Lancet. 1999 Mar 20;353(9157):1000–1.

10. Alvarez GG, VanDyk DD, Aaron SD, Cameron DW, Davies N, Stephen N, et al. Taima (stop) TB: the impact of a multifaceted TB awareness and door-to-door campaign in residential areas of high risk for TB in Iqaluit, Nunavut. PLoS ONE. 2014;9(7):e100975.

11. Minich K, Saudny H, Lennie C, Wood M, Williamson-Bathory L, Cao Z, et al. Inuit housing and homelessness: results from the International Polar Year Inuit Health Survey 2007-2008. Int J Circumpolar Health. 2011;70(5):520–31.

12. Huet C, Ford JD, Edge VL, Shirley J, King N, IHACC Research Team, et al. Food insecurity and food consumption by season in households with children in an Arctic city: a cross-sectional study. BMC Public Health. 2017 15;17(1):578.
